# Loss of ADAR1 protein induces changes in small RNA landscape in hepatocytes

**DOI:** 10.1261/rna.080097.124

**Published:** 2024-09

**Authors:** Kristina Roučová, Václav Vopálenský, Tomáš Mašek, Edgar del Llano, Jan Provazník, Jonathan J.M. Landry, Nayara Azevedo, Edvard Ehler, Vladimír Beneš, Martin Pospíšek

**Affiliations:** 1 Laboratory of RNA Biochemistry, Department of Genetics and Microbiology, Faculty of Science, Charles University, 128 00 Prague, Czech Republic; 2 Laboratory of Biochemistry and Molecular Biology of Germ Cells, Institute of Animal Physiology and Genetics, CAS, 277 21 Liběchov, Czech Republic; 3 GeneCore Facility, EMBL, 69117 Heidelberg, Germany; 4 Department of Biology and Environmental Studies, Faculty of Education, Charles University, 116 39 Prague, Czech Republic

**Keywords:** RNA editing, ADAR1, miRNA, snoRNA, Y RNA, hepatocyte

## Abstract

In recent years, numerous evidence has been accumulated about the extent of A-to-I editing in human RNAs and the key role ADAR1 plays in the cellular editing machinery. It has been shown that A-to-I editing occurrence and frequency are tissue-specific and essential for some tissue development, such as the liver. To study the effect of ADAR1 function in hepatocytes, we have created Huh7.5 ADAR1 KO cell lines. Upon IFN treatment, the Huh7.5 ADAR1 KO cells show rapid arrest of growth and translation, from which they do not recover. We analyzed translatome changes by using a method based on sequencing of separate polysome profile RNA fractions. We found significant changes in the transcriptome and translatome of the Huh7.5 ADAR1 KO cells. The most prominent changes include negatively affected transcription by RNA polymerase III and the deregulation of snoRNA and Y RNA levels. Furthermore, we observed that *ADAR1* KO polysomes are enriched in mRNAs coding for proteins pivotal in a wide range of biological processes such as RNA localization and RNA processing, whereas the unbound fraction is enriched mainly in mRNAs coding for ribosomal proteins and translational factors. This indicates that ADAR1 plays a more relevant role in small RNA metabolism and ribosome biogenesis.

## INTRODUCTION

A-to-I type of editing is performed by adenosine deaminases acting on RNA (ADARs). By deaminating adenosine residues, inosine (I) is introduced to RNA. The replacement of an amino group by an oxo group changes the hydrogen bond acceptor and donor sites, affecting thus both the RNA sequence and nucleotide base-pairing. There are three human ADAR proteins out of which only two are catalytically active, ADAR1 and ADAR2. ADAR1 is responsible for the majority of A-to-I editing outside the brain. A shorter variant of the ADAR1 protein (p110) is expressed constitutively and ubiquitously and localizes mainly in the cell nucleus. A longer variant (p150) is expressed upon interferon (IFN) stimulation and localizes in the cytoplasm (Patterson and Samuel 1995). ADAR1 protein is composed of two Z-DNA binding domains, three dsRNA-binding motifs, and one deaminase domain. Through its binding and editing activity, ADAR1 can influence miRNA processing, alternative splicing, nuclear export, degradation, or protection of RNA molecules as reviewed in Wang et al. (2017). Besides its catalytic activity, ADAR1 can influence cellular processes by interacting directly with other proteins in the cell, like Dicer (Ota et al. 2013) and PKR (Clerzius et al. 2009). Sheer dsRNA-binding activity of ADAR1 was also shown to protect mRNA from the Staufen1-mediated mRNA decay (Sakurai et al. 2017).

A huge effort has been put into identifying sites subjected to ADAR1 editing. Using the fact, that inosine preferentially pairs as guanosine, edited sites can be identified through sequencing as A-to-G mismatches. With the rise of high-throughput methods, most studies adopted the bioinformatic approach of aligning the whole genome and transcriptome sequences (Bahn et al. 2012; Peng et al. 2012; Ramaswami et al. 2013). This concentrated endeavor revealed that the distribution and level of ADAR1-dependent RNA editing is tissue-specific and the vast majority of ADAR1-dependent editing occurs in Alu repetitive sequences (Levanon et al. 2004; Kiran and Baranov 2010; Ramaswami and Li 2014; Picardi et al. 2017). Furthermore, the profile of A-to-I editing was observed to differ in various types of cancer at both the mRNA and miRNA levels (Mingardi et al. 2018; Tassinari et al. 2019; Heraud-Farlow and Walkley 2020). Being inducible by IFN, ADAR1 also serves as a part of the cellular antiviral machinery and can edit viral RNA and influence its splicing (Liu et al. 2015; Tomaselli et al. 2015; Figueroa et al. 2016; Pfaller et al. 2018).

Various types of small RNAs have been discovered up to now, and some of them have been also documented as ADAR1 targets. miRNAs are short noncoding RNAs performing a posttranscriptional regulation of gene expression. The primary transcript folds back on itself and forms a hairpin structure, which is processed by enzymes DROSHA and Dicer into a 21–23 nt long dsRNA molecule. Then, one of the strands is loaded onto RISC to perform its gene expression regulation. Although *ADAR1* overexpression has been observed to have little effect on the global miRNA expression (Chen et al. 2015; Ishiguro et al. 2018), ADAR1 can bind (Ishiguro et al. 2018) and alter expression of certain miRNAs (Galipon et al. 2017; Cho et al. 2018; Yujie et al. 2020) and/or their processing to mature miRNAs (Díaz-Piña et al. 2018). ADAR1 can also edit miRNAs including their seed regions, which can affect their pairing ability and target binding specificity (Heale et al. 2009; Nigita et al. 2016).

Small nucleolar RNA (snoRNA) are another type of small RNAs in the cell. They are canonically involved in the maturation of rRNA and snRNA in the nucleolus, but can bind other targets like lncRNA or even mRNA and influence their splicing (Watkins and Bohnsack 2012). ADAR1 has been shown to be localized in the nucleolus as well, although it is believed to be catalytically inactive there (Desterro et al. 2003; Vitali et al. 2005). However, this might be challenged because nucleolar RNAs are enriched in Alu repeat elements scattered within intronic sequences, which are required together with nucleolin for maintaining nucleolus structure and targeting genomic loci to the nucleolus (Caudron-Herger et al. 2015). A snoRNA-related lncRNA has been shown to promote ADAR1 dimerization and increase its A-to-I editing activity recently (Huang et al. 2022). Deeper connection between ADAR1 protein and snoRNA has not been made yet.

Y RNAs are evolutionarily conserved and can be found in all animals but also in some bacteria and archaea. They associate with Ro60 protein and form together a core of Ro60 RNPs. Ro60 RNPs play presumably an important role in the quality control of noncoding RNAs, including pre-5S rRNA and U2 snRNAs (Hendrick et al. 1981). Y RNA can function also independently of Ro60 as has been shown in DNA replication (Christov et al. 2006). Ro60 was also reported to function as a repressor of ADAR1-mediated editing of Alu sequences (Quinones-Valdez et al. 2019). However, functions of Y RNAs and Ro60 RNPs in human cells are largely unexplored and most of the molecular and structural data are derived from bacteria.

So far, ADAR1 has been studied in the human cells mainly through big sequencing projects of various tissues and in cell lines using knockdown assays. Viable *ADAR1* knockouts (KO) were gained only in HEK293T and HeLa cell lines (Pestal et al. 2015; Pfaller et al. 2018). Another attempt included knocking out *ADAR1* in human embryonic stem cells and their subsequent differentiation into hepatocyte-like cells or neuronal progenitors (Chung et al. 2018). In our study, we strived to identify changes that the loss of ADAR1 induces in differentiated human hepatocytes, because the liver and hepatocytes specifically belong to the most affected tissues in *ADAR1* KO mice (Hartner et al. 2004; Wang et al. 2015). We aimed for a nonembryonic differentiated hepatocyte cell line Huh7.5, which is a broadly used model cell line and is permissive for hepatotropic viruses such as hepatitis C virus and hepatitis B virus (Blight et al. 2002; Le et al. 2021). We used this new Huh7.5 ADAR1 KO cell line to test whether ADAR1 loss influences mRNA loading to polysomes. Our analysis revealed that other types of small RNAs are also influenced by ADAR1 loss, namely, snoRNAs and Y RNA, which have not yet been reported in connection to ADAR1 to the best of our knowledge.

## RESULTS

### Generation of Huh7.5 *ADAR1* KO cell lines

**FIGURE 1.**
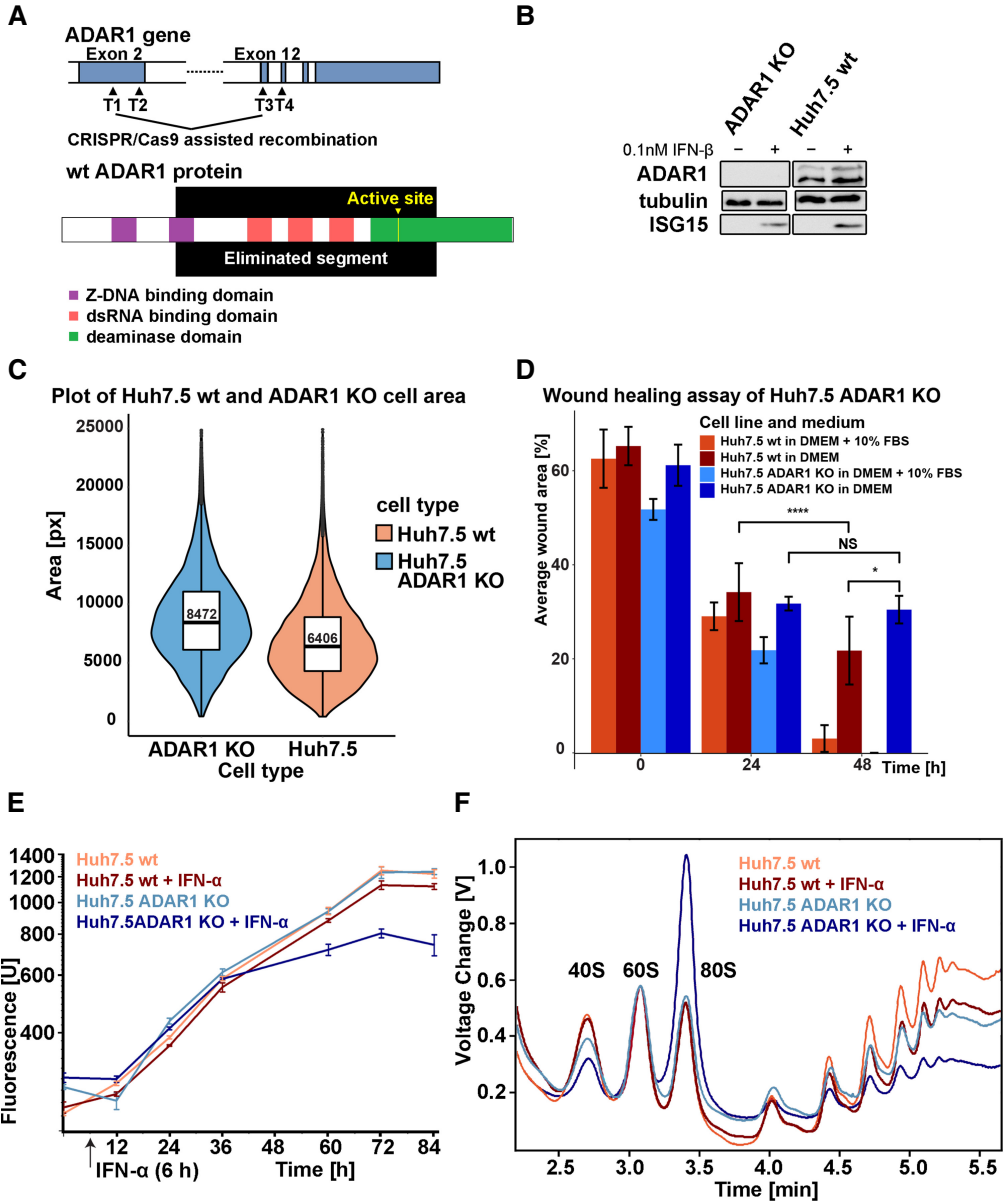
Characterization of Huh7.5 *ADAR1* KO phenotype. (*A*) Schematic depiction of CRISPR/Cas9-directed *ADAR1* gene recombination in Huh7.5 cell line. The recombination between target sequences (marked T1–T4) in the region coding exon 2 and exon 12 of *ADAR1* mRNA is designed to eliminate a significant portion of the *ADAR1* gene. The wild-type (wt) variant of the ADAR1 protein is shown with its functional domains: two Z-DNA binding domains (purple), three dsRNA-binding domains (pink), and deaminase domain (green), including the active site (yellow). The recombination eliminates all three dsRNA-binding domains and the majority of the deaminase domain with the active site. The depiction is based on the longest transcript variant of *ADAR1*, ID: NM_001111.4. (*B*) Western blot analysis of *ADAR1* KO efficiency. Huh7.5 wt and Huh7.5 ADAR1 KO cell lines were grown for 24 h in DMEM + 10% FBS with or without 0.1 nM IFN-β up to full confluence and harvested. Lysate samples containing an equal amount of proteins (40 µg) were used for the western blot analysis. The complete loss of ADAR1 bands (both for p110 and p150) in the Huh7.5 ADAR1 KO cell line can be observed as well as the preservation of ISG15 expression upon IFN treatment. *ADAR1* KO was also confirmed by PCR and sequencing of the target region. (*C*) Quantification of size increase of Huh7.5 ADAR1 KO cells compared to Huh7.5 wt cells using fluorescent microscopy. We stained both cell types with WGA (membrane) and DAPI (nuclei) and used this staining to calculate the area covered by individual cells. The graph combines a violin plot, which shows the frequency of cell areas measured for Huh7.5 wt (red) and Huh7.5 ADAR1 KO (blue), and a box plot, which shows the first quartile, median, and the third quartile. The median values for each cell line are stated in the box plot. The graph shows that Huh7.5 ADAR1 KO cells area distribution is shifted to larger sizes. The quantification method is described in the Materials and Methods section. (*D*) Wound healing assay of Huh7.5 wt and Huh7.5 ADAR1 KO cells. The initial wound was done just to a confluent monolayer of Huh7.5 wt and Huh7.5 ADAR1 KO cells. The graph shows the size of the wound at 0, 24, and 48 h (*n* = 6, significance of differences was assessed by paired and two-sample *T-*test). In the presence of FBS, Huh7.5 wt and Huh7.5 ADAR1 KO cells healed the wound at a similar rate. Without FBS, the wound in Huh7.5 ADAR1 KO monolayer stayed bigger than in Huh7.5 wt; NS *P* > 0.05; (*) *P* < 0.05; (****) *P* < 0.0001. (*E*) Growth properties of Huh7.5 wt and Huh7.5 ADAR1 KO cells. The growth was measured by the resazurin assay. Both cell types were grown in a regular medium (DMEM + 10% FBS). IFN was added 6 h postseeding. In a regular medium (DMEM + 10% FBS), the growth rates for Huh7.5 wt (red) and Huh7.5 ADAR1 KO (blue) were similar. Huh7.5 ADAR1 KO cells stopped their growth as soon as 24 h upon IFN addition and died by 72 h (dark blue). Error bars for each data point show the standard deviation of the triplicate measured. (*F*) Polysome profile analysis of the Huh7.5 wt and the derived ADAR1 KO cells under normal and IFN conditions. Polysomes were analyzed from cells upon 24 h of 0.1 nM IFN-α treatment. Data are normalized to 60S peak. There is a shift from the polysomal fraction in favor of the ribosomal 80S peak in the IFN-treated Huh7.5 ADAR1 KO (dark blue) compared to IFN-treated Huh7.5 wt (dark red).

We used a CRISPR/Cas9 system to generate an ADAR1-deficient cell line from a differentiated hepatoma cell line Huh7.5. In total, we designed four gRNAs to target the *ADAR1* gene. Two gRNAs target the start and the end of exon 2 (ADAR_Targ1 and ADAR_Targ2), one targets exon 12 (ADAR_Targ3), and one targets exon 13 (ADAR_Targ4) (Fig. 1A; Materials and Methods). Our aim was to achieve a large recombination and eliminate a significant portion of the *ADAR1* gene, therefore impairing the expression of both p110 and p150 variants. We successfully tested our CRISPR/Cas9 system on the HEK293 cell line, a parental cell line of HEK293T, in which a viable elimination of ADAR1 was reported previously (Supplemental Fig. S01; Pestal et al. 2015). After optimization of the system for the Huh7.5 cell line, we successfully obtained Huh7.5 *ADAR1* KO and confirmed the recombination event by sequencing and western blot analysis (Fig. 1B). The recombination in the *ADAR1* gene in the Huh7.5 cell line occurred before ADAR_Targ1 and ADAR_Targ3 sites. About 15.4 kbp of the *ADAR1* gene was excised by the recombination, which led to the elimination of all three dsRNA-binding domains and half of the catalytic domain, including the active site of the enzyme (Fig. 1A).

### Huh7.5 ADAR1 KO cells display altered morphology and profound response to interferon

Morphologically, the Huh7.5 ADAR1 KO cells are similar to their wt parent (Supplemental Fig. S02). When working with Huh7.5 ADAR1 KO cells, we observed an increase in apparent cell size when the cells were adherent to the surface of the culture dish. We quantified this parameter for Huh7.5 wt and Huh7.5 ADAR1 KO cells by measuring the area of fluorescently labeled cells adherent to a surface and the particle size of trypsinized cells in suspension. The area covered by the Huh7.5 ADAR1 KO cell on the dish (median = 8472 px) was larger than the area covered by the Huh7.5 wt cell (median = 6406 px). The average (median) Huh7.5 ADAR1 KO cell was 32% larger than its wt counterpart, when it grew adherent to the dish surface (Fig. 1C). When converted to a cell suspension by trypsinization, both cell lines showed basically the same cell size of ∼18 µm as determined by the cell counter (Supplemental Table S01).

A cell size increase was previously observed for Huh7 cells growing in human serum (HS) supplemented medium (Steenbergen et al. 2013). We tested if Huh7.5 wt and ADAR1 KO cells get larger in HS supplemented medium or if the cell size increase for the Huh7.5 ADAR1 KO cells is final. We obtained volunteer male and female HS. Adherent Huh7.5 ADAR1 KO cells increased their area by 72% and 43% after their passaging for 12 weeks in media supplemented with female and male HS, respectively. Adherent wt Huh7.5 cells increased their area only by 31% and 22%, respectively, in a parallel experiment (Supplemental Table S02).

Observed differences in surface area size between the Huh7.5 wt and Huh7.5 ADAR1 KO adherent cells while keeping their volume similar suggested possible differences in their interaction with the dish surface and prompted us to assess their migration capabilities. To analyze that, we performed a wound healing assay in normal and serum-free medium. In normal medium, Huh7.5 ADAR1 KO cells healed the wound at a similar rate as Huh7.5 wt. In serum-free medium, after 24 h both cell types slowed their migration rate, although Huh7.5 ADAR1 KO did so more rapidly. In the latter case, the wound never healed completely (Fig. 1D).

To further characterize both cell lines, we measured the growth rate of the newly established Huh7.5 ADAR1 cell line using a resazurin-based approach. Under normal conditions, growth rates of KO cells and wt were comparable with average division times 28.91 h for Huh7.5 wt and 29.23 h for Huh7.5 ADAR1 KO (Supplemental Table S03). However, there was a significant difference in their response to the IFN-α treatment. Whereas Huh7.5 wt cells merely slightly decreased their growth rate, Huh7.5 ADAR1 KO cells stopped growth and died within 3 days after IFN-α treatment (Fig. 1E). Polysome profiling showed a rapid decrease of polysomal fraction in favor of a monosome peak in the Huh7.5 ADAR1 KO cell line after IFN-α treatment (Fig. 1F). This is in a good agreement with a previously observed translational shutdown in HEK293T ADAR1 KO cells (Chung et al. 2018). However, we observed the translational shutdown as soon as 24 h after IFN-α/β application in Huh7.5 ADAR1 KO cells, thus substantially earlier than 48 h, which was reported for HEK293T ADAR1 KO (Fig. 1E,F; Supplemental Fig. S03; Chung et al. 2018).

To check IFN signaling pathway integrity in the Huh7.5 ADAR1 KO cell line, we analyzed the ISG15 levels in the cells cultivated in normal medium and in medium with added IFN. We used IFN-α or IFN-β in concentrations as low as 0.1 nM to induce ISG15 expression. Western blot analysis showed that ISG15 induction by IFN was not impaired in cells depleted of ADAR1 (Fig. 1B). In some experiments, the western blot showed a slight ISG15 production even without IFN addition in Huh7.5 ADAR1 KO cells compared to Huh7.5 wt cells (Supplemental Fig. S04). This could be due to autocrine IFN signaling of the Huh7.5 ADAR1 KO cells. A similar trend of local inflammation was observed in the liver of ADAR1 KO mice (Hartner et al. 2004; Wang et al. 2015).

### ADAR1 influences mRNA abundance in the cell

**FIGURE 2.**
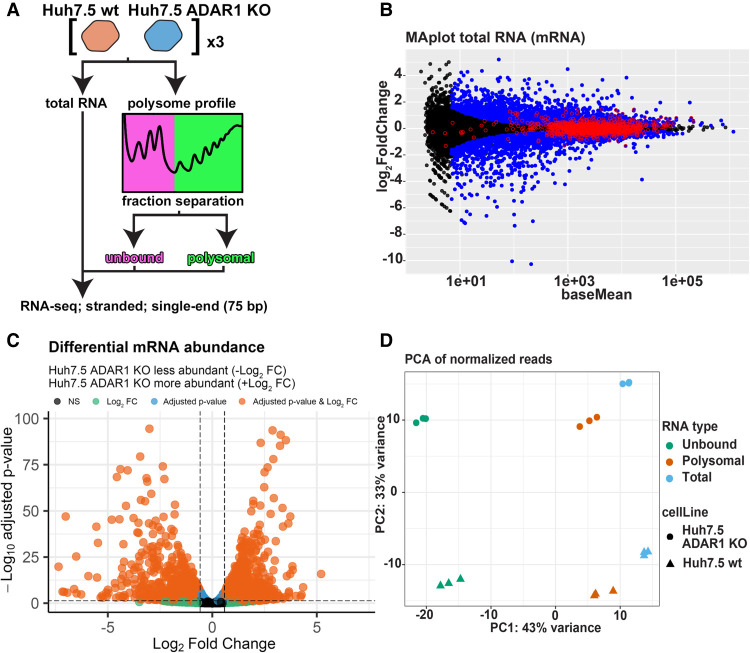
RNA-seq and differential expression in RNA samples of Huh7.5 wt and Huh7.5 ADAR1 KO. (*A*) Schematic depiction of analyzed RNA types. Total RNA and RNA from the polysome profiles were isolated from both Huh7.5 wt and Huh7.5 ADAR1 KO cell lines. The polysome profiles were dissected in two fractions: all from the loading peak up to the monosomal 80S peak (unbound) and the rest (polysomal). All six sample types were subjected to RNA-seq analysis. (*B*) MA plot of DESeq2 results for mRNAs in total RNA samples. The plot shows results of differential expression analysis done by DESeq2. Significantly changed genes (FC > 1.5, *P-*adj < 0.05) are in blue, unchanged genes are in black. Edited genes are marked with a red circle. (*C*) Volcano plot of differential mRNA abundance. The plot shows DESeq2 results for mRNA changes in total RNA samples. Threshold for FC is set to 1.5, and threshold for adjusted *P*-value is set to 0.05. Genes passing both thresholds are in orange, genes passing only the FC threshold are in green, genes passing only the adjusted *P*-value are in blue, genes not passing any threshold are in gray. (*D*) Principal component analysis of all mRNA sequencing samples. Counts for the DESeq2 analysis were normalized by the DESeq2 rlog. In the plot, along the PC1 axis, different mRNA types can be separated (unbound, polysomal, and total), and along the PC2 axis, the different cell lines can be separated (Huh7.5 wt and Huh7.5 ADAR1 KO).

Findings about the effect of ADAR1 loss on mRNA abundance differ. In HEK293T cells, it was reported that lack of ADAR1 protein does not influence transcript abundance in the cell (Chung et al. 2018), whereas others found hundreds of differentially expressed transcripts (Hartner et al. 2009; Liddicoat et al. 2015). The influence of ADAR1 protein on transcript abundance might be cell type-specific and might be specifically high in hepatocytes because the liver is substantially more affected in *ADAR1* KO mice than other tissues (Hartner et al. 2009; Liddicoat et al. 2015; Wang et al. 2015). Therefore, we isolated and sequenced total RNA from Huh7.5 wt and Huh7.5 ADAR1 KO human hepatocellular carcinoma cells (Fig. 2A; Supplemental Fig. S05A). Using the DESeq2 package, the differential expression analysis showed 1308 genes with increased expression and 1018 genes with decreased expression (with fold change [FC] > 1.5 and adjusted *P*-value < 0.05) (Fig. 2B,C). This is in agreement with previous findings of other groups using *ADAR1* KO mice as a model (Hartner et al. 2009; Liddicoat et al. 2015; Pestal et al. 2015). We validated our DESeq2 results by performing RT-qPCR on randomly selected genes with prominent increase and/or decrease in mRNA abundance determined by FC in the DESeq2 analysis. The results of RT-qPCR were consistent with the DESeq2 results (Supplemental Fig. S06).

We performed a gene set enrichment analysis (GSEA) on the DESeq2 results data set devoid of genes with not applicable adjusted *P*-value to assess which biological processes are, namely, influenced by ADAR1 depletion in the Huh 7.5 cells. We used the WEB-based GEne SeT AnaLysis Toolkit (WEBGESTALT) (Liao et al. 2019). The analysis showed an enrichment of categories involved in the number of biological processes (FDR < 0.05), mainly amine metabolic process, nucleoside bisphosphate metabolic process, fatty acid derivative metabolic process, neutral lipid metabolic process, and fatty acid metabolic process (NES, normalized enrichment score > 2) (Supplemental Table S04). Similar analysis using the molecular function GO database demonstrated increased expression of genes from categories monooxygenase activity, lipid transporter activity, oxidoreductase activity, and acting on CH—OH group of donors (NES > 2) (Supplemental Table S04). Apparently, the expression of genes involved in processes which are typical for hepatocytes is among the most affected by the *ADAR1* KO. On the other side of the spectrum, leading among the biological process categories with negative enrichment scores were transcription by RNA polymerase III (RNA Pol III) and RNA splicing (NES < −2). In accordance with the latter, GSEA with the cellular compartment gene data set revealed decreased expression of genes belonging to the spliceosomal complex category (NES < −2) (Supplemental Table S04).

### Loss of ADAR1 affects mRNA loading into polysomes

We wanted to test whether loss of ADAR1 can influence the translation of individual mRNAs. To achieve that, we decided to analyze the distribution of individual mRNAs between pools of polysome bound and polysome unbound cellular mRNAs. We performed a polysome profile analysis from Huh7.5 wt and Huh7.5 ADAR1 KO cells and divided the profiles into two different fractions (Fig. 2A). The first fraction contained the initial part of the profile, including the peak corresponding to the 80S ribosome (further named “unbound”). The second fraction contained the remaining part of the profile comprising polysome peaks (further named “polysomal”). Poly(A)+ RNAs from these fractions were sequenced, and the RNA-seq data were analyzed using DESeq2 to investigate how ADAR1 loss influences their distribution along the polysome profile (Supplemental Fig. S05B,C). To assess the variance between different sample types, we performed a principal component analysis of the RNA-seq data (PCA). PCA showed that the most profound differences between samples could be attributed to their localization along the polysome profile and to the cell line of origin (Fig. 2D).

**FIGURE 3.**
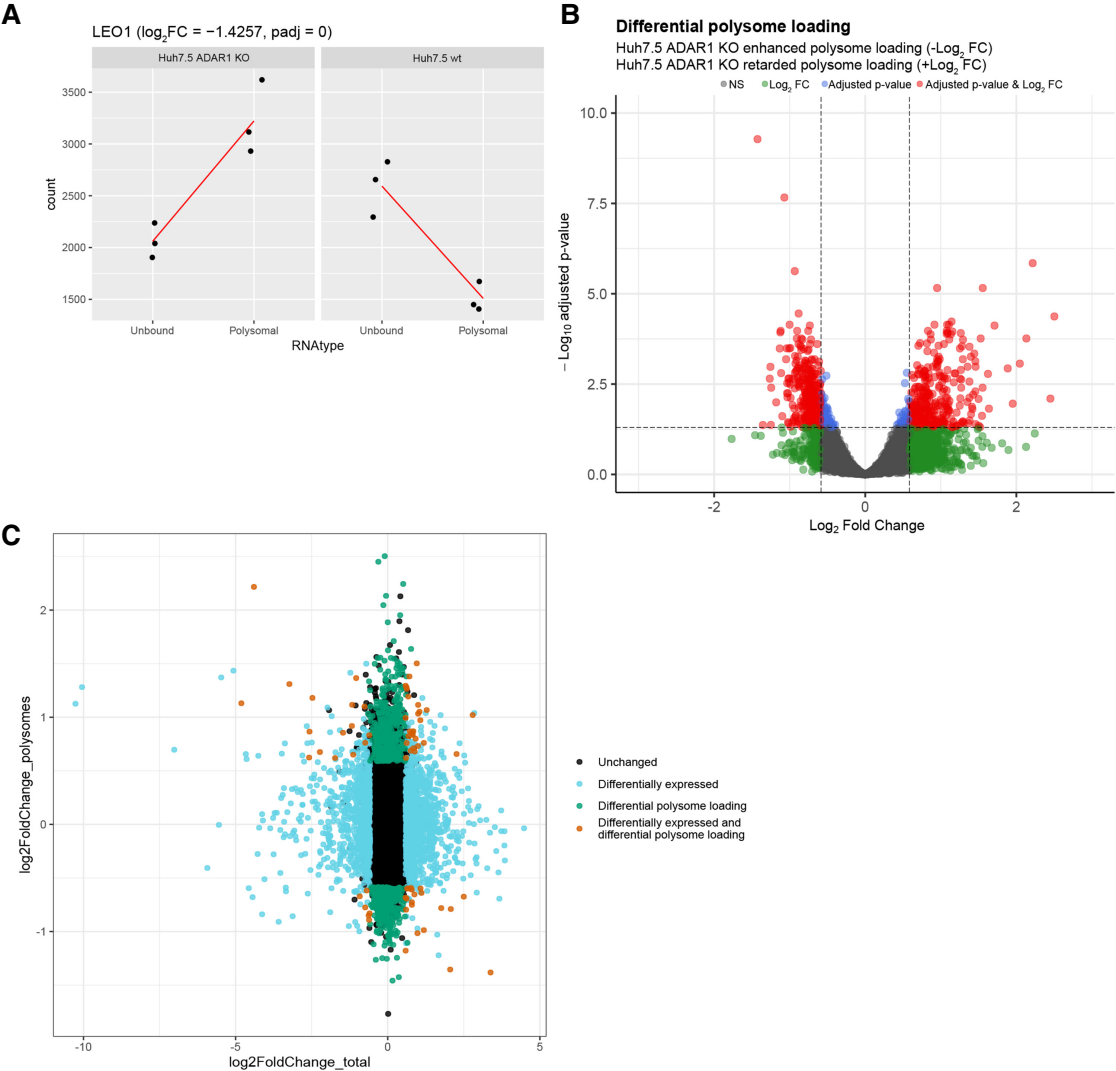
Differential analysis of Huh7.5 ADAR1 KO and Huh7.5 wt translatomes. (*A*) Schematic depiction of mRNA differential expression and polysome loading analysis. DESeq2 analysis was done with interaction design of mRNA type and cell line parameters for Huh7.5 ADAR1 KO and wt cells. As an example, we show the normalized read counts for the *LEO1* gene that exhibits increased polysome loading in Huh7.5 ADAR1 KO cells in comparison to Huh7.5 wt polysomes (log_2_FC = −1.425749). Further examples of mRNA behavior leading to differential polysome loading are depicted in Supplemental Figure S07. (*B*) Volcano plot of differential mRNA loading onto polysomes. The plot shows DESeq2 results for mRNA changes in total RNA samples. Threshold for FC is set to 1.5 and threshold for adjusted *P*-value is set to 0.05. Genes passing both thresholds are in red, genes passing only the FC threshold are in green, genes passing only the adjusted *P*-value are in blue, genes not passing any threshold are in gray. (*C*) Scatter plot of differentially expressed genes and genes with differential polysome loading. Out of 18,366 genes from total RNA analysis, only 13,835 genes that also appear in the differential polysome loading analysis are shown. Differentially expressed genes (FC > 1.5 and adjusted *P*-value <0.05 in total RNA) are in blue, genes with differential polysome loading are in green, genes that are both differentially expressed and exhibit differential polysome loading are in red, remaining genes are in black (unchanged).

To compare mRNA partitioning between the unbound and polysome fraction in *ADAR* KO and wt cells, we implemented the DESeq2 interaction analysis between the two parameters (cell line and mRNA source fraction). This enabled us to observe cell line-specific changes in mRNA distribution between the two parts of the polysome profile (unbound and polysome fractions) (Love et al. 2014). In other words, it allowed us to compare the relative differential abundance of transcripts in unbound and polysomal fractions with respect to the cell line of origin and suppress possible influence of differences in particular mRNAs abundances in total RNA pools of both compared cell lines (Fig. 3A). Further examples of this analysis are depicted in Supplemental Figure S07. Using this approach, we calculated log_2_(fold change) parameter (log_2_FC), negative value of which corresponds to mRNAs with enhanced polysome loading in Huh7.5 ADAR1 KO cells or retarded mRNA polysome loading in Huh7.5 wt cells. The other way around, its positive value corresponds to retarded mRNA polysome loading in Huh7.5 ADAR1 KO cells and enhanced polysome loading in Huh7.5 wt cells (examples in Fig. 3A; Supplemental Fig. S07). Even though it is not always true, if we place an equal sign between the increased mRNA loading into polysomes and enhanced translation of these particular mRNAs, then we can simplify that in this kind of analysis −log_2_FC corresponds to an enhanced translation of the particular mRNA in Huh7.5 ADAR KO cells in comparison with Huh7.5 wt.

We identified 318 genes exhibiting retarded translation and 335 genes exhibiting enhanced translation in the Huh7.5 ADAR1 KO cell line in comparison with its parental Huh7.5 wt cells (FC over 1.5 and adjusted *P*-value < 0.05) (Fig. 3B; Supplemental Table S05). There were 66 pseudogenes, mainly pseudogenes of ribosomal proteins, enriched in the retarded translation gene group. GSEA analysis of mRNAs demonstrating differential translation between Huh7.5 wt and Huh7.5 ADAR KO cells revealed that loss of ADAR1 led to a relative increase in the representation of a substantial number of categories in the biological process GO database (Supplemental Table S06). Categories with NES higher than 2.0 representing mRNAs with retarded translation in Huh7.5 ADAR1 KO cells comprise the following biological processes (BP): protein localization to the endoplasmic reticulum, translational initiation, establishment of protein localization to the membrane, cytoplasmic translation, protein targeting, and RNA catabolic process. Categories with NES lower than −2.0 representing mRNAs with enhanced translation in Huh7.5 ADAR1 KO cells include the following biological processes: protein localization to chromosome, RNA localization, regulation of chromosome organization, tricarboxylic acid metabolic process, chromosome segregation, microtubule cytoskeleton organization involved in mitosis, RNA polyadenylation, DNA conformation change, actin filament-based movement, mRNA processing, toxin transport, spindle organization, and microtubule anchoring. Similarly, for the cellular compartment database, categories with NES < −2 include nuclear periphery, preribosome, microtubule organizing center part, chromosomal region, transcription elongation factor complex, chaperone complex, Cajal body, site of DNA damage; category with NES > 2 was ribosome, and cytosolic part. For the molecular function database, categories with NES < −2 comprise helicase activity, ATPase activity, histone binding, ligase activity, RNA polymerase binding, motor activity, oxidoreductase activity, acting on the aldehyde or oxo group of donors, tubulin binding, ADP binding, modification-dependent protein binding, whereas categories with NES > 2 were structural constituents of ribosome and rRNA binding. Interestingly, if we count GO categories with the NES below −1.8 and above 1.8 in the biological processes GO category, we receive a significantly higher number of categories with NES < −1.8. This result could be interpreted that ADAR1 KO led to the overall increase in global cellular translation and to the deregulation of many biological processes in the hepatocellular carcinoma cells. Similar observation comes from the GSEA analysis using the cellular compartment and molecular function GO data sets (Supplemental Table S06).

When we take a look at the genes and categories that appear in both RNA abundance analysis (total RNA) and polysome loading analysis (unbound vs. polysomal fraction comparisons) (Fig. 3C), we can observe that transcripts of the category protein localization to the endoplasmic reticulum (GO:0070972) with retarded translation in Huh7.5 ADAR1 KO are also more abundant in the Huh7.5 ADAR1 KO cell line (Supplemental Table S07). On the other hand, mRNAs in categories exhibiting enhanced translation in Huh7.5 ADAR1 KO cells are less abundant in the *ADAR1* KO cell line: mRNA processing (GO:0006397), RNA localization (GO:0006403), RNA splicing (GO:0008380), protein-containing complex localization (GO:0031503), ribonucleoprotein complex localization (GO:0071166), and positive regulation of signaling receptor activity (GO:2000273) (Supplemental Table S07).

We noticed that the unbound fraction of the Huh7.5 ADAR1 KO cells is significantly enriched in transcripts of genes containing snoRNA genes within their locus. We tested our DESeq2 data set if snoRNA coding is an aspect that more generally influences gene enrichment in the retarded translation group. Interestingly, we found that transcripts of genes containing snoRNA genes within their locus had more than 8.7 times higher odds to be in the Huh7.5 ADAR1 KO retarded translation group than genes without snoRNA genes (logistic regression, *P* < 2 × 10^−16^).

### A-to-I editing does not influence the target mRNA abundance or its translation behavior

One of the obvious possible explanations of differential expression or translation in Huh7.5 ADAR1 KO cell lines is the lack of ADAR1 editing. To examine this, we used JACUSA software (Piechotta et al. 2017) to identify ADAR1 edited positions in the Huh7.5 cell line. JACUSA software enables the comparison of the cDNA sequences directly without the need of comparing sequences to the reference genome first.

We compared data from the triplicates of total RNA-seq from Huh7.5 wt and Huh7.5 ADAR1 KO cells. Using RNA-seq data from the Huh7.5 ADAR1 KO cell line is advantageous because the results are thus not influenced by the residual ADAR1 activity as has to be expected in more common studies based on *ADAR1* knockdown. Also, comparing the KO cell line to its parental cell line minimizes the influence of SNPs. After filtering (see Materials and Methods), we obtained 8664 positions that were edited only in Huh7.5 wt samples and not in Huh7.5 ADAR1 KO samples corresponding to 1082 unique genes with annotated RefSeq transcripts (O'Leary et al. 2016). In total, 1062 of genes with edited transcripts were present in our DESeq2 results (Supplemental Table S08).

**FIGURE 4.**
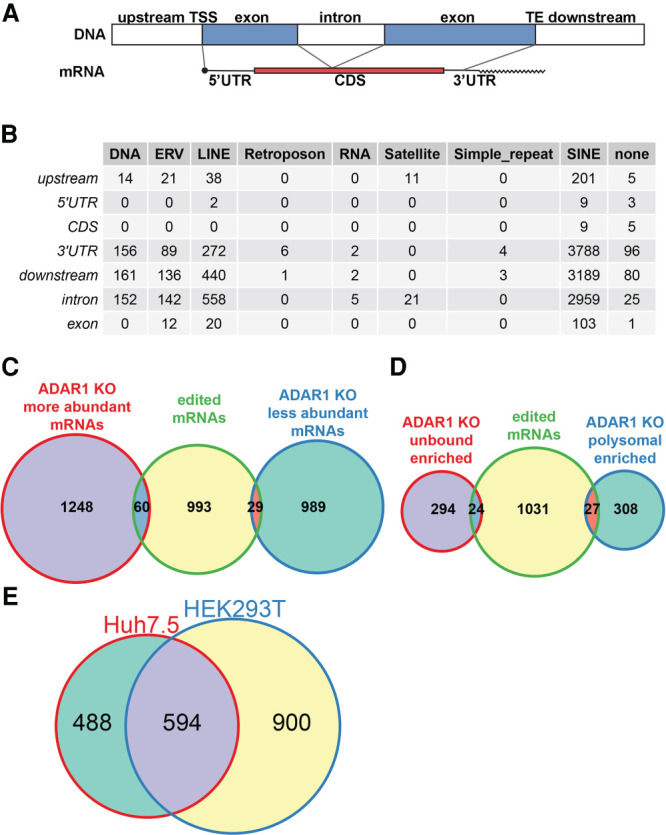
Edited position and their corresponding genes in Huh7.5 cell line. (*A*) A schematic depiction of used RNA/gene part annotations. On the *top*, there is the DNA composition of a hypothetical gene. Positions located between a database given gene start and a transcription start site (TSS) are annotated as “upstream.” Analogically, positions located after a database given TE up to the gene end are annotated as “downstream.” Annotations for “5′ UTR,” “CDS,” “3′ UTR,” and “intron” follow the database annotations. For transcripts without UTR and CDS annotations, a simple “exon” category was established. BiomaRt (version 2.38.0) was used to assign annotations. (*B*) A table of edited sites identified in Huh7.5 ADAR1 KO to Huh7.5 wt total RNA comparison. The table summarizes all identified edited positions based on their position in the transcript and the type of repetitive element they are part of. Repetitive elements were assigned to positions using Repbase v. 23.11. Positions not located in a database annotated repetitive element are grouped in “none” column. (*C*) Venn diagram of edited mRNA abundance. The diagram shows number of edited mRNAs belonging to unique genes which are differentially abundant in Huh7.5 ADAR1 KO cells compared to Huh7.5 wt. (*D*) Venn diagram of edited mRNA polysome loading. The diagram shows number of edited mRNAs belonging to unique genes which exhibit altered polysome loading in Huh7.5 ADAR1 KO cells in comparison to Huh7.5 wt. (*E*) Venn diagram of unique genes with edited transcripts in Huh7.5 and HEK293T cell lines. The diagram shows that about half of the genes with edited transcripts in Huh7.5 overlap with the set of genes with edited transcripts in HEK293T identified by Chung et al. (2018).

We classified A-to-I edited nucleotide positions into categories based on their location within the RefSeq transcripts (Fig. 4A) and the repetitive element type assigned to that location in Repbase (v23.11) (Bao et al. 2015). We assigned two extra categories “upstream” and “downstream” for edited positions located between a BioMart database given gene start and a transcript start and positions located after a database given transcript end (TE) up to gene end, respectively (biomaRt attributes start_position, transcript_start, transcript_end, and end_position, respectively) (Smedley et al. 2015). These positions may correspond to a 5′ or 3′ UTR of another transcript of the gene, but for our analysis, we considered only RefSeq transcripts. The results show that most of the edited positions were located in the 3′ UTRs and introns of transcripts containing SINE elements. Only a few A-to-I edited sites were detected in the mRNA coding sequences (Fig. 4B). Inosine can pair with C, A, and U during decoding in ribosomes or can induce a ribosome stalling (Licht et al. 2019). Even though we applied only the simplest I-to-G decoding rule, we found possible nonsynonymous changes in the polypeptide chains caused by A-to-I editing in transcripts of the following genes: *SRP9*, *ZNF669*, *C11orf80*, *TROAP*, *COG3*, *CLTC*, *ZNF587B*, *FLNB*, *NOP14*, and *H2BC5.* Detailed information about the exact position of the edited site, editing frequency, possible changes in the corresponding polypeptide chain, and comparison with data in REDIportal and ADeditome databases (Mansi et al. 2021; Wu et al. 2021) are described in Supplemental Table S09. The positions edited in coding regions of *CLTC*, *ZNF91*, and *H2BC5* in Huh7.5 cell lines are not proposed to be A-to-I edited in neither REDIportal nor ADeditome databases.

We wanted to check if lack of the ADAR1-dependent A-to-I editing can be correlated with mRNA abundance. In the group of 1308 genes that were more abundant in total RNA from the Huh7.5 ADAR1 KO cell line (FC > 1.5, adjusted *P*-value < 0.05), there were only 60 edited mRNAs passing our set filters (Fig. 4C; Supplemental Table S08). Out of the 1018 less abundant mRNAs, 29 gene transcripts were edited. Using logistic regression, we found that the edited transcripts have decreased odds to be differentially expressed between Huh7.5 ADAR1 KO and Huh7.5 wt cells (Table 1).

**TABLE 1.** Logistic regression of the chance of edited transcripts to belong among the differentially abundant and/or differentially translated mRNAs

Huh7.5 ADAR1 KO group of	Odds of edited transcript to belong to group	*P*-value
more abundant transcripts	0.75	0.0383
less abundant transcripts	0.45	3.54e-05
retarded translation	0.96	0.854
enhanced translation	1.03	0.869

Further, we found 24 edited transcripts with retarded translation and 27 edited mRNAs with enhanced translation in Huh7.5 ADAR1 KO cells (Fig. 4D; Supplemental Table S08). Behavior of these mRNAs in translation does not correlate with the lack of editing (Table 1).

We compared our list of edited positions for the Huh7.5 cell line with the list provided for HEK293T by Chung et al. (2018), who identified 1494 unique A-to-I edited transcripts in the HEK293T transcriptome. Transcripts of 594 genes were shared between their and our set of A-to-I edited mRNAs (Fig. 4E). We took our and Chung's lists of genes with edited transcripts and performed an overrepresentation analysis (ORA) using the WebGestalt tool (Liao et al. 2019). The analysis revealed that genes present in both sets belong, besides others, to the significant number of enriched categories involved in RNA processing and metabolism (Supplemental Table S10). The majority of the categories are shared between the HEK293T and Huh7.5 data sets analyzed by ORA individually (Supplemental Table S11).

### Lack of ADAR1 leads to changes in the abundances of small RNAs

**FIGURE 5.**
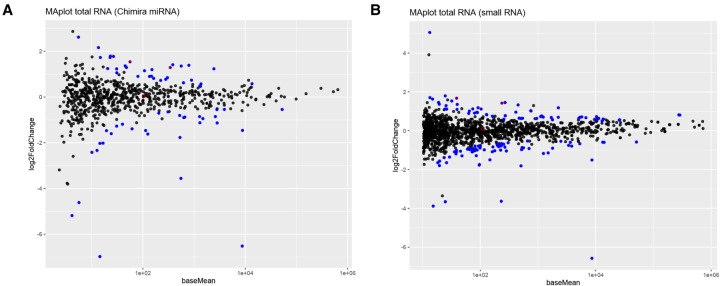
miRNA and small RNA changes in Huh7.5 ADAR1 KO. (*A*) MA plot of DESeq2 results for miRNAs mapped by Chimira in total RNA samples. The plot shows results of differential expression analysis done by DESeq2. Significantly changed miRNAs (FC > 1.5, *P-*adj < 0.05) are in blue, unchanged miRNAs are in black. Edited miRNAs are marked with a red circle. (*B*) MA plot of DESeq2 results for small RNA sequencing mapped to the genome in total RNA samples. The plot shows results of differential expression analysis done by DESeq2. Significantly changed small RNAs (FC > 1.5, *P-*adj < 0.05) are in blue, unchanged small RNAs are in black. Edited small RNAs are marked with a red circle.

As mentioned above, we found that mRNAs of genes harboring snoRNA genes within their transcription unit have a higher probability to demonstrate retarded translation in Huh7.5 ADAR1 KO cells. Therefore, we specifically sequenced small RNAs contained in total RNA, unbound, and polysome preparations from both Huh7.5 wt and Huh7.5 ADAR1 KO cells. We identified 702 miRNAs using the Chimira web tool (Vitsios and Enright 2015) in the total RNA preparation and analyzed their levels by DESeq2 (Fig. 5A; Supplemental Fig. S08A). Twenty-eight unique miRNAs were more abundant in total RNA preparation from Huh7.5 ADAR1 KO cells, and 28 miRNAs were less abundant (Fig. 5A; Supplemental Table S12). In the unbound versus polysomal fraction comparison analogical to the mRNA DESeq2 analysis, we found three miRNAs enriched in the Huh7.5 ADAR1 KO unbound fraction and 12 miRNAs enriched in the Huh7.5 ADAR1 KO polysomal fraction. To assess if the changed miRNA abundance correlates with their target abundance, we combined these findings with the list of miRNA targets from miRTarBase (version 7.0) (Huang et al. 2019). In our case, we limited the list only to targets supported by strong evidence in the database. Most miRNAs correspond to many mRNA targets in miRTarBase and consequently also to many mRNAs among the gene transcripts significantly detected in Huh7.5 ADAR1 KO cells. We attempted to analyze these data by various ways, including analysis of principal component. However, we were unable to detect any clear common correlation between differential expression of miRNAs in total RNAs of Huh7.5 wt and Huh7.5 ADAR1 KO cells and their targets' abundance or distribution alongside the polysome profile. The data are summarized in Supplemental Table S12. Similar analysis of the most prominently changed hsa-mir-10b led to the same conclusion in the sense of counts of positively and negatively changed target mRNAs even when including its weak miRTarBase targets in the analysis. Nevertheless, we can speculate about the possible contribution of increased abundance of some hsa-mir-10b targets including *FUT6, FUT1*, and *KLF4* to the observed morphological changes of Huh7.5 ADAR1 KO cells (Supplemental Table S13).

We checked if any of the miRNAs in our data are edited specifically by ADAR1. We found miRNAs hsa-mir-9903, hsa-mir-3144, hsa-mir-625, and hsa-mir-561 to contain an edited nucleotide. For hsa-mir-9903 and hsa-mir-561, the guide strand is edited, for hsa-mir-3144 and hsa-mir-625, the passenger strand is edited. Interestingly all of these miRNAs are edited within the 2–7 nt of the 5′ region, which could potentially change their target binding preferences, mainly in those edited in the guide strand. To investigate this, we used the miRNA target prediction web tool of miRDB (Chen and Wang 2020). The edited and unedited variants of miRNAs control almost completely separate sets of targets (Supplemental Fig. S09). Out of the edited miRNA, only miRNA hsa-mir-625 and hsa-mir-561 exhibit increased abundance in the total Huh7.5 ADAR1 KO RNA samples, specifically miR-625-3p and miR-561-5p, which are the edited strands. The hsa-mir-3144, hsa-mir-625, and hsa-mir-561 would be classified as Huh7.5 ADAR1 KO unbound fraction enriched, but the adjusted *P*-value did not pass the filtering (adjusted *P*-value < 0.05).

We analyzed the abundance and polysome loading of mRNAs targeted by edited miRNAs. However, we were unable to detect any clear correlation between the editing of miRNAs and their target abundance or distribution alongside the polysome profile. The data are summarized in Supplemental Table S14.

We did not observe any change in the abundance or the polysome loading behavior of miRNA processing genes *DROSHA*, *DGCR8*, *DICER1*. *AGO1*, *AGO2*, and *PACT*. Only *TRBP* exhibited retarded polysome loading in Huh7.5 ADAR1 KO cells.

Because Chimira annotates reads only to a miRNA database (miRBase 22.1) (Kozomara et al. 2019), we decided to map the reads from small RNA sequencing also to the whole-genome reference (GRCh38) to assess possible changes in other small cellular RNAs (Fig. 5B; Supplemental Fig. S08B). Our alignment method revealed 41 more abundant small RNAs (17 miRNAs, 24 snoRNA, or *SNHG*) and 37 less abundant small RNAs (28 miRNAs, 8 RNY or RNYP, 1 scaRNA) in total RNA preparation of the Huh7.5 ADAR1 KO cell compared to Huh7.5 wt cells (Supplemental Table S15). From the groups of more and less abundant miRNAs, 16 and 20 miRNAs from direct mapping to the genome, respectively, were shared between our and Chimira mapping. Furthermore, our mapping allowed us to see changes in other small cellular RNAs, like the increased abundance of snoRNA and decreased abundance of RNY and their pseudogenes in the Huh7.5 ADAR1 KO cell line. When we compared the abundance of certain read lengths sequenced in wt and Huh7.5 ADAR1 KO cells, we noticed that some read lengths are enriched or underrepresented in Huh7.5 ADAR1 KO sequencing. By performing size-specific read alignment, we again found less abundant RNY, namely, RNY1, RNY4, and RNY3 at sizes 30 and 37 nt in Huh7.5 ADAR1 KO total RNA preparation. These might correspond to RNY fragments found in apoptotic (Rutjes et al. 1999) or proliferating cells (Nicolas et al. 2012). In polysome analysis, eight small RNAs were enriched in the Huh7.5 ADAR1 KO polysomal fraction (seven miRNAs, one scaRNA).

## DISCUSSION

With the increasing number of *ADAR1* knockdown and KO studies, it becomes obvious that the effects of the lack of ADAR1 or its overexpression are both heavily influenced by the parental tissue type of the cells used (Giacopuzzi et al. 2018). This indicates the involvement of not only ADAR1 itself, but also the specific mechanisms and pathways that activate after the cell differentiation and are thus specific for the given cell type. The liver has been shown to be among the most affected organs upon ADAR1 loss (Hartner et al. 2004). Although, attempts have been made to establish human hepatic cells lacking ADAR1 protein (Chung et al. 2018; Liu et al. 2019), to our knowledge a complete KO has not yet been presented. In this study, we successfully obtained a viable ADAR1 KO cell line of differentiated hepatocytes Huh7.5 and investigated changes induced by such an alteration.

Morphologically, the Huh7.5 ADAR1 KO cells do not display many changes, which have been shown also for the human iPS cells and HEK293T (Katayama et al. 2015; Chung et al. 2018). We observed an increase in the Huh7.5 ADAR1 KO cell size compared to the Huh7.5 wt, which has recently been shown also for cardiomyocytes, where ADAR1 depletion led to the enlargement of the whole heart and increased the likelihood of a fatal heart failure (El Azzouzi et al. 2020). So far, ADAR1 loss in the liver has been reported to reduce the liver size, which is, however, attributed to cell death (Hartner et al. 2004). Huh7.5 ADAR1 KO proliferation also appears unchanged as it was shown for hESC (Chen et al. 2015). The response to IFN was much faster in Huh7.5 ADAR1 KO than it was reported for HEK293T ADAR1 KO cells. The onset of decreased growth could be observed as soon as 24 h after IFN-α addition, which is substantially sooner than the 48 h that were previously reported for HEK293T ADAR1 KO cells (Chung et al. 2018). Huh7.5 ADAR1 KO are also substantially more sensitive to IFN treatment than HEK293 ADAR1 KO and HEK293T ADAR1 KO cell lines. To obtain a marked decrease in growth and global translation, a 10 times higher concentration of IFN has to be applied to HEK293 ADAR1 KO and HEK293T ADAR1 KO cell lines than to Huh7.5 ADAR1 KO (Fig. 1E,F; Supplemental Fig. S03; Chung et al. 2018). Same as for the HEK293T, the Huh7.5 ADAR1 KO cells underwent translational arrest induced by IFN from which they did not recover. We observed specific low induction of ISG15 in Huh7.5 ADAR1 KO cells (Supplemental Fig. S04), which supports the previously reported induction of IFN signaling accompanying ADAR1 deficiency (Hartner et al. 2009; El Azzouzi et al. 2020) and may help to explain the observed inflammation of the liver tissue in ADAR1-deficient mice (Hartner et al. 2004). A failure to regulate the response to autosecreted IFN in ADAR1 KO hepatic cells might be the cause for their cell growth arrest and death (Wang et al. 2015). A-to-I editing destabilizes dsRNA structures. ADAR1 loss thus increases the structural integrity of cellular dsRNAs and leads to chronic activation of cellular dsRNA sensors, including PKR, RIG-I, MDA5, and OASes, which may lead to various pathogenic states, such as Aicardi–Goutières syndrome and tumorigenic inflammation (Chen and Hur 2022).

Creating the Huh7.5 ADAR1 KO cell line enabled us to identify edited positions specific for human hepatocytes. While we still have to keep in mind that Huh7.5 is a cancer-derived cell line and its editing profile could be altered from normal hepatocytes, we clearly showed that groups of gene transcripts edited in Huh7.5 and HEK293T overlap only partially. Part of the edited transcripts identified in Huh7.5 belongs to categories strongly associated with the liver (e.g., lipid homeostasis) (Kawahara et al. 2007). Consistent with other studies, we found that positions edited by ADAR1 in the Huh7.5 cell line are particularly present in the noncoding regions of 3′ UTR and introns, mostly Alu repeats (Cho et al. 2018). However, we also identified 13 A-to-I edited positions in CDS that in five cases led to silent or conservative missense changes in mRNA. This is valid for the most expected interpretation of inosine as guanosine. However, inosine can form pairs and be decoded also with adenosine and uridine or can induce a ribosome stalling as has been evidenced recently (Licht et al. 2019). Interestingly, two of the proteins with edited coding mRNA sequences are involved in cell adhesion (trophinin) and connection of the cell membrane to the actin cytoskeleton (filamin-B). Some of the other proteins are involved in protein modification, transport, and secretion (Signal recognition particle 9 kDa protein, Conserved oligomeric Golgi complex subunit 3, Clathrin heavy chain, Peptidylprolyl isomerase like 3 and Rhomboid domain-containing protein 3) (Stelzer et al. 2016). Disbalance in the production of cell adhesion-related proteins may contribute to the observed differences between Huh7.5 wt and Huh7.5 ADAR1 KO cells in their cell-surface size and ability to heal wounds. Irregularities in the production of part of these could also influence the function of the protein secretion pathway and consequently may lead to observed prominent downregulation of translation of mRNAs coding for proteins involved in protein localization to the endoplasmic reticulum. Nucleolar protein 14 (NOP14) is among the few proteins possibly affected by ADAR1 editing. NOP14 plays a role in pre-18S rRNA processing and small ribosomal subunit assembly (Stelzer et al. 2016). Possible alterations in these processes may also indirectly contribute to the observed significant changes in polysome loading of mRNAs coding for proteins classified into the rRNA binding and preribosome and ribosome part GO categories. A-to-I editing has been detected also in coding mRNA sequences for three zinc finger proteins, from which ZNF91 is specifically required to repress SINE-VNTR-Alu (SVA) retrotransposons (Stelzer et al. 2016). In this case, I interpretation as G leads to synonymous codon reading; however, other base pairs and/or ribosome stalling cannot be excluded (Stelzer et al. 2016).

ADAR1 has two major ways it can influence gene expression. The first is through its editing activity where ADAR1 can alter the RNA sequence and influence its processing, coding potential, translatability, and stability (Wang et al. 2017). Secondly, it can influence the gene expression by its sheer RNA binding capability (Heale et al. 2009; Sakurai et al. 2017). Furthermore, this is not valid just for mRNAs, but also for miRNAs, which in turn can again influence gene expression at the level of transcript translation or degradation (Yang et al. 2006; Heale et al. 2009; Ota et al. 2013; Yates et al. 2013; Nigita et al. 2016).

Loss of ADAR1 protein has been shown to have a big influence on mRNA abundance in cells (Hartner et al. 2009; Liddicoat et al. 2015), which is consistent with our findings of hundreds of differentially expressed genes in Huh7.5 ADAR1 KO cells. We and others also did not observe a significant correlation between RNA editing events and mRNA abundance (Heale et al. 2010; Nakano et al. 2016; Guallar et al. 2020). On the other hand, several studies proposed that while the loss of ADAR1 influences heavily the protein level, the mRNA level remains unchanged (Yang et al. 2017). Few differentially expressed genes and no significant differential enrichment of any gene sets were reported in the transcriptome comparison between WT and ADAR1-deficient HEK293T cells (Chung et al. 2018). Our data do not provide any evidence about the correlation between A-to-I editing of transcripts and their loading and/or unloading onto polysomes (Fig. 4D; Table 1).

Two of the four miRNAs, which we identified as edited, show increased abundance in Huh7.5 ADAR1 KO, but we did not observe a consistent effect on their mRNA targets (Supplemental Table S14). All four edited miRNAs had a relatively low abundance and the A-to-I editing frequency of the edited nucleotide was only ∼20% for those included in miRTarBase (hsa-mir-3144, hsa-mir-625, and hsa-mir-561). Individual miRNAs can recognize hundreds of mRNA targets and most of their binding sites are noncanonical. It has been shown that even more vigorous experiments based on either overexpression or knockdown of cognate miRNAs typically led to only small changes in the expression of their individual targets which were further difficult to reconcile with any phenotype. The assumed role of the miRNA regulatory network is thus rather maintaining homeostasis of gene expression and reducing its noise than functioning as its prominent on–off switch (Hausser and Zavolan 2014; Schmiedel et al. 2015). Edited miRNAs identified by us in human hepatocytes thus could broaden the regulatory network of their cognate miRNAs which may rather lead to the difficult-to-catch small changes in the global gene expression homeostasis than to the well-observable perturbations in the abundances of the individual mRNA targets.

We observe a higher abundance of the aryl hydrocarbon receptor mRNA in Huh7.5 ADAR1 KO total RNA, which was shown to be related to miR-378 target site loss in ADAR1 KD Huh7 cells (Nakano et al. 2016). Editing of AHR mRNA is in our data indeed lost, yet we do not observe a shift in the AHR mRNA polysome loading. miR-378 abundance itself is in our data unchanged.

The most differentially decreased miRNA in Huh7.5 ADAR1 KO cells is miR-10b (Supplemental Table S12). Higher levels of miRNA-10b are associated with increased metastasis, increased migration, and increased invasive potential in many cancer types, including hepatocellular carcinoma in which elevated levels of miR-10b can also serve as a negative prognostic marker (Sheedy and Medarova 2018; Aksoy et al. 2022). Among the most differentially increased miR-10b target mRNAs in Huh7.5 ADAR1 KO are fucosyltransferases *FUT1* and *FUT6* and Krüppel-like transcription factor *KFL4* (Supplemental Table S13). All these proteins have been found to play a role in cell adhesion, migration, and tumor invasion (Kanoh et al. 2003; Palumberi et al. 2010; Desiderio et al. 2015; Lai et al. 2019; He et al. 2023). It is tempting to speculate that the decrease of miR-10b contributes to observed differences between wt and *ADAR* KO human hepatocytes in their cell-surface area size and behavior in wound healing assay.

Analysis of mRNA distribution between polysomes and unbound fractions enabled us to identify a set of transcripts of ribosomal protein pseudogenes to be enriched in the *ADAR1* KO unbound fraction. Unlike the common opinion of pseudogenes as junk DNA, they are often transcribed and number of reports about their possible biological function, including protein coding and/or serving as a source of functional noncoding RNA, is increasing (Cheetham et al. 2020). Considering global changes in mRNA loading to polysomes and affected ribosome synthesis in Huh7.5 ADAR1 KO cells, further investigation of the possible connection between ribosomal protein pseudogenes and ADAR1 function could be meaningful. Besides editing and differential expression of some miRNA, we also observed the deregulation of some other small RNAs, which might be even more important for the cell physiology. To our knowledge, the ADAR1 influence on small RNAs other than miRNAs remains largely unexplored. We observed that the loss of ADAR1 protein led to an increase in the abundance of several snoRNAs. This may directly affect ribosome biogenesis and cause a decreased plasticity in ribosome composition change upon stress, including stimulation by IFN. This would be certainly a direction worth exploring.

Analysis of small RNA content in Huh7.5 ADAR1 KO hepatocytes revealed a decreased abundance of Y RNAs. We did not observe any changes in their interaction partner Ro60 mRNA abundance or its loading to polysomes. One of the suggested functions of Y RNAs is the regulation of Ro60 protein activity in noncoding RNA quality control (Boccitto and Wolin 2019). Therefore, decreased levels of Y RNAs might be one of the causes of the observed deregulation of ribosome biogenesis and misbalanced levels of some snoRNAs in Huh7.5 ADAR1 KO cells. Y RNAs are transcribed by RNA Pol III, and their decreased levels in *ADAR1* KO hepatocytes are in a good agreement with the observed decrease of RNA Pol III transcription upon ADAR1 depletion (Supplemental Fig. S10). ADAR1 has been found together with ADAR2 in the nucleolus (Desterro et al. 2003); however, unlike ADAR2, no specific function has been assigned to ADAR1 in this cellular compartment, yet. We found a significant decrease of mRNAs coding for several RNA Pol III subunits and transcription factors in Huh7.5 ADAR1 KO cells (Supplemental Table S04), which may suggest a direct way for investigation into the ADAR1 role in the nucleolus. Interestingly there are contradictory reports about ADAR1 role in A-to-I editing of Alu elements transcribed by RNA Pol III. In HeLa cells, these Alu transcripts were edited by ADAR1 (Dupuis 2012), whereas in HEK293T no A-to-I editing of the RNA Pol III Alu transcripts was observed (Chung et al. 2018). We show here that transcription of RNA Pol III subunits and transcription factors are significantly affected by ADAR1 depletion, which also has to be taken into account for future analysis of A-to-I editing of RNA Pol III-transcribed RNAs. Regulation of RNA Pol III and thus Y RNA expression by ADAR1 provides also an unexpected piece to the puzzle of onset of autoimmune diseases such as systemic lupus erythematosus and Sjögren's syndrome in which the role of Ro60 and ADAR has been proposed (Boccitto and Wolin 2019; Dolcino et al. 2019; Quinones-Valdez et al. 2019; Muro et al. 2020).

Besides analysis of the whole transcriptome including small and micro RNAs, we also analyzed translatome in Huh7.5 ADAR KO cells and compared it with the Huh7.5 wt translatome. To do that, we developed a new approach that simultaneously considers trends in mRNA distribution between polysomes and the unbound fraction in Huh7.5 wt and *ADAR* KO cells and thus allows us to pick up a set of transcripts enriched either in polysomes or in the unbound fraction of both Huh7.5 wt and Huh7.5 ADAR1 KO cells. This analysis revealed significant differential enrichment of mRNAs coding for ribosomal proteins, translation factors, and proteins involved in protein targeting and localization in the unbound fraction of Huh7.5 ADAR1 KO cells (or in other words, in polysomes of Huh7.5 wt). Six of the nine biological process GO categories significantly enriched in mRNAs in the Huh7.5 ADAR1 KO unbound fraction are somehow associated with translation and/or protein targeting. Opposite to this, mRNAs belonging to 77 GO biological process categories were differentially enriched in Huh7.5 ADAR1 KO polysomes and/or in the Huh7.5 wt unbound fraction (Supplemental Table S06). However, the total number of mRNAs enriched in the unbound fraction is roughly comparable with those enriched in the polysomal fraction in Huh7.5 ADAR1 KO cells (Fig. 3B). This disproportion may mean that a generally higher fraction of key mRNAs classified into distinct biological process GO categories is differentially loaded to Huh7.5 ADAR1 KO polysomes than to the unbound fraction. This may mean increased translation of these mRNAs as well as a slower flow of these mRNAs through polysomes and their retardation on translating ribosomes. The latter might be more probable because of the possibly affected ribosome synthesis in Huh7.5 ADAR1 KO cells. Deeper investigation will be needed to better understand this phenomenon.

The differences between the observed impact of ADAR1 deficiency on changes in mRNA levels and editing of Alu transcripts across different studies are both probably cell line-specific. The HEK293T ADAR1-deficient cell line revealed negligible changes in gene expression and no A-to-I editing of Pol III-transcribed Alu elements (Chung et al. 2018). On the other hand, both ADAR1- and ADAR2-mediated editing of Pol III-transcribed Alu elements were found in HeLa cells (Dupuis 2012). Similarly, we found a large number of differentially expressed genes, including decreased expression of several RNA Pol III subunits and general transcription factors, between the Huh7.5 wt cell line and its ADAR1-deficient derivative. One of the reasons could be the that whereas HeLa and Huh7.5 cell lines were derived from the fully differentiated somatic cells, HEK293T probably originated from the immature embryonic neuronal cells (Shaw et al. 2002). Another difference, which may play a role, could be the overall level of the A-to-I editing activity in the parental cells. This can be reproducibly measured and expressed with the help of the Alu editing index (AEI) (Roth et al. 2019). Schaffer et al. (2020) determined AEI in 1610 cell lines and found that only 52 of them displayed higher editing activity than the median AEI value corresponding to 1.14. HEK293T cells have been used for testing transiently expressed ADARs due their low intrinsic ADAR activity. HEK293T AEI is lower (0.63 as inferred from Roth et al., Fig. 4), than AEI of their parental cell line HEK293 (1.069) and Huh7 (1.206) (Roth et al. 2019; Schaffer et al. 2020). High intrinsic A-to-I editing activity in the Huh7 cell line may also explain our observed difficulties in preparation of *ADAR1* KO in its derivative Huh7.5 in comparison with the HEK293 where preparation of *ADAR1* KO was relatively easy. The HEK293T cell line has been immortalized by transfection of the adenovirus serotype 5 DNA and further improved by expression of the SV40 large T antigen. Both adenoviral proteins and SV40 large T antigen heavily influence the transcription machinery of all eukaryotic polymerases including RNA Pol III as well as impact cellular antiviral response (Sollerbrant et al. 1993; Larminie et al. 1999; Samuel 2012; Price et al. 2022; UniProt Consortium 2023). Adenovirus type 5 also strongly stimulates the transcription of endogenous Alu elements by RNA Pol III (Panning and Smiley 1993). The presence of adenoviral proteins and the SV40 large T antigen could thus be the reason for the low differential gene expression between HEK293T and its ADAR1-deficient derivative and for the undetectable A-to-I editing in the RNA Pol III-transcribed Alu elements observed previously (Chung et al. 2018).

## MATERIALS AND METHODS

### Cell lines

The Huh7.5 cell line (human, male) was kindly provided by C.M. Rice based on an MTA. Cell lines were maintained in regular Dulbecco's modified Eagle's medium (DMEM, Sigma-Aldrich), with 10% inactivated fetal bovine serum (FBS), at 37°C in a humidified atmosphere containing 5% CO_2_ and were passaged regularly.

### Plasmid construction

Gene-specific targets were designed using ZiFiTTargeter (version 4.2) (http://zifit.partners.org/ZiFiT/Disclaimer.aspx). A pair of reverse complementary oligonucleotides (1 µL of 100 µM of each primer; marked as Top and Bot; see Supplemental Table S16) were annealed (95°C for 6 min; 50°C for 6 min; 37°C for 60 min, and 22°C for 180 min) in 1× concentrated T4 ligation buffer in a total volume of 20 µL and phosphorylated by the addition of 2U T4 Polynucleotide Kinase (Fermentas) and 2 µL of 1 mM ATP (Sigma-Aldrich) for 80 min at 37°C. After phosphorylation, a particular annealed duplex was cloned into a dephosphorylated (Shrimp alkaline phosphatase—Fermentas) BbsI digested pU6-sgRNA vector (Shan et al. 2013), using T4DNA ligase (Fermentas). To prepare the pRR-Puro recombination-dependent reporter (Flemr and Bühler 2015) containing sequences homologous to the targeted gene, we mixed all the duplexes of a particular gene together, treated them with a Klenow fragment of DNA polymerase I (4 µL of phosphorylated duplexes, 5U of a Klenow fragment [Fermentas], 1 µL of 2 mM dNTPs [Roche] at 37°C for 25 min, froze for 120 min at −20°C), treated them with T4 DNA ligase (Fermentas) for 180 min at 22°C, and ligated them into an Ecl136II linearized and Shrimp alkaline phosphatase (Fermentas) dephosphorylated pRR-Puro plasmid using T4DNA ligase. All clones were verified by PCR analysis, restriction endonuclease digestion, and sequencing.

### Cell culture transfection

Cells were transfected by Lipofectamine 2000 Transfection Reagent (Invitrogen), according to the manufacturer's instructions. In short, cells were seeded in a 24-well plate overnight to reach 80% confluence at the time of transfection; 1.5 µL of Lipofectamine 2000 Transfection Reagent was mixed with a total of 1 µg of plasmid DNA in 50 µL of Opti-MEM Medium, incubated at room temperature for 5 min and added to cells. One day after transfection, the medium was changed for fresh DMEM + 10% FBS. Two days after transfection, the medium was changed for DMEM containing 10% FBS and supplemented with 2 µg/mL Puromycin (Sigma-Aldrich). After 1 day of cultivation, cells were detached from the dish surface by trypsin, resuspended in an equal volume of Puromycin-containing medium, and cultivated for 4 days. The medium was then changed for regular DMEM containing 10% FBS. After 9 days, grown colonies were transferred to a 96-well plate and tested for recombination.

### Cell growth assay

Cells were freshly grown and seeded into a 96-well plate at 20%–30% confluence. For the IFN parallels, normal medium was changed for medium containing 2 µg/mL IFN-β (Sino Biologicals) or IFN-α (Sino Biologicals) 6 h after seeding cells. With the exception of the 0 h point, the medium was changed before resazurin (Sigma-Aldrich) addition. Ten percent of the well medium volume of resazurin was added to each measured well, and cells were incubated at 37°C in a humidified atmosphere containing 5% CO_2_. After 4 h of incubation, the medium was transferred into a black 96-well plate, and fluorescence was measured using Varioskan Flash (Thermo Fisher Scientific), excitation 530 nm, emission 585 nm, bandwidth 5 nm for 100 msec.

### Cell size assay

Cells were seeded in a 6-well plate containing a microscopy cover glass at a density to reach ∼70% confluence after overnight cultivation. The following day, cells were washed with PBS (Lonza) and fixed by 4% formaldehyde for 15 min at 37°C. Cells were washed three times with Hank's Balanced Salt Solution (HBSS, 0.14M NaCl, 5 mM KCl, 1 mM CaCl_2_, 0.4 mM MgSO_4_, 0.5 mM MgCl_2_, 0.3 mM Na_2_HPO_4_, 0.4 mM KH_2_PO_4_, 4 mM NaHCO_3_, and 6 mM glucose). Stock fluorescein-labeled (FITC) Wheat Germ Agglutinin (WGA, Vector Laboratories) was diluted to a working concentration 5.0 µg/mL in HBSS. Cells were labeled at room temperature for 10 min. Labeling solution was aspired and cells were washed twice with HBSS. Cells were permeabilized by 0.2% Triton X-100 in HBSS for 3 min. Cover glass was then mounted on a glass slide with Mowiol (Polysciences) containing 0.1 µg/mL DAPI. Slides were then analyzed by fluorescent microscopy on a Nikon Ti2.

Pictures of 100 (10 × 10) consecutive fields were taken in the green (FITC) and blue (DAPI) channels. Images were sewn together, and the resulting picture was used for analysis in MATLAB. The nuclei count was determined by separate DAPI signals with a desired roundness and intensity to exclude dividing or dying cells. The nuclei were then used for a region-growing algorithm across the green signal. Data for all cell areas obtained this way are shown in Figure 1C.

### Wound healing assay

Cells were grown to confluence in a 6-well plate. A wound was made to the monolayer by scratching the surface with a pipette tip. Pictures of the wound were taken at 24 and 48 h postscratching. The size of the wound was evaluated in ImageJ. The significance of different healing speeds was assessed by paired or two-sample *T*-test (*n* = 6).

### Western blot analysis

The medium was aspired from the culture, and cells were lysed directly using a 2× loading buffer (125 mM Tris-Cl, 3% SDS, 10% gylcerol, 0.01% bromphenol blue, and 3.3% β-mercaptoethanol). Lysates were boiled for 10 min prior to loading on 12% SDS PAGE Tris–Gly gel. Proteins were blotted to a PVDF membrane (Bio-Rad) and probed for ADAR1 (Sigma-Aldrich), ISG15 (Santa Cruz), and tubulin (Abcam). Blots were developed using chemiluminescent detection. All antibodies used for western blot analysis are listed in Supplemental Table S16.

### Polysome profiling

The polysome profiling method has been described elsewhere (Masek et al. 2020). To summarize, cells were grown to 80% confluence and then treated with 100 µg/mL cycloheximide for 5 min at 37°C. From that point on, cells were handled on ice. Cells were washed with cold PBS with 100 µg/mL cycloheximide and lysed in a polysome profile lysis buffer (10 mM HEPES [pH 7.5], 62.5 mM KCl, 5 mM MgCl_2_, 2 mM DTT, 1% Triton X-100, 100 μg/mL cycloheximide, Complete EDTA-free [Roche, 1 tablet/10 mL], and 40 U/mL Ribolock [Thermo Scientific]). Lysates were incubated on ice for 20 min with occasional vortexing. Lysates were centrifuged at 8000*g* for 5 min at 4°C. RNA content of lysates was measured using Nanodrop. One milligram of RNA was cast on a 10%–50% sucrose gradient, which was prepared in solution containing 10 mM HEPES (pH 7.5), 100 mM KCl, 5 mM MgCl_2_, 2 mM DTT, 100 μg/mL cycloheximide, complete EDTA-free (1 tablet/100 mL), and 5 U/mL Ribolock (Thermo Scientific). Gradients were prepared in Gradient Master 108 v5.3 (Biocomp). Gradients were centrifuged in an SW41Ti rotor at 35,000*g* for 3 h in an Optima L-90 Ultracentrifuge (Beckman Coulter). Polysome profiles were obtained using an ISCO UA-5 detector and ISCO UV absorbance reader (Teledyne, ISCO); data were collected and processed using Clarity Lite software (DataApex).

### RNA extraction

Total RNA was isolated from freshly grown cells in 10 cm dishes using TriReagent (Sigma-Aldrich). Cells were washed with PBS and covered by 1.2 mL of TriReagent. To augment cell lysis, cells were scraped from the dish surface. Lysates were transferred into a tube and vortexed; 350 µL of chloroform was added for RNA extraction. The mixture was vortexed and centrifuged for 20 min at 13,000*g* and 4°C. The RNA-containing fraction was transferred into a clean tube, and RNA was precipitated by adding 1× volume of isopropanol, vortexing, and incubating at −20°C for 1 h. RNA precipitate was pelleted by centrifugation and washed twice with 75% ethanol. RNA was then air dried and diluted in RNase-free water. RNA yield and quality were assessed via agarose electrophoresis and Nanodrop measurement.

Polysome profile fractions, unbound and polysomal, were collected directly during polysome profile analysis. Fractions were mixed with 1× volume of 5.25 M guanidine thiocyanate and 0.25 M natrium citrate, vortexed and mixed with 1.33× volume of isopropanol and incubated at −20°C overnight. Samples were centrifuged at 15,000*g* and 4°C for 40 min and washed with 75% ethanol. Precipitate was air dried and dissolved in TriReagent; 0.35× volume of chloroform was added and mixture thoroughly vortexed. After 25 min of centrifugation at 13,500*g* and 4°C, the RNA-containing phase was transferred into a new tube, and 1 µL of GenElute-LPA (Sigma-Aldrich) was added. RNA was then precipitated by adding 1× volume of 75% ethanol and incubating at −20°C overnight. Precipitate was washed twice with 75% ethanol, air dried and dissolved in RNase-free water. RNA yield and quality was assessed via agarose electrophoresis and Nanodrop measurement.

### RT-qPCR

Five micrograms of RNA from each sample was reverse-transcribed using 20 U of M-MuLV Reverse Transcriptase (Thermo Scientific), and 1 µg of oligo(dT) primer in a reaction volume of 40 µL. cDNA synthesis was performed at 37°C for 5 min followed by incubation at 42°C for 75 min and subsequent inactivation at 70°C for 10 min. qRT-PCR experiments were performed using a LightCycler 480 (Roche) and LightCycler 480 SYBR Green I Master mix (Roche). The 10 µL reactions were performed in triplicates. Each reaction contained 2 µL of cDNA and 500 nM gene-specific primers (list of used primers is provided in Supplemental Table S16). The amplification protocol was 95°C for 5 min; 44 cycles of 95°C for 10 sec, 58°C for 15 sec, 72°C for 15 sec; followed by melting curve determination.

### Library preparation and RNA sequencing

Libraries for mRNA sequencing were prepared with the TruSeq Stranded mRNA kit (Illumina) using 250 ng of RNA per library. Samples were prepared in three biological replicates for each cell line (Huh7.5 wt and Huh7.5 ADAR1 KO) and RNA type (total, unbound, and polysomal). mRNA was isolated from total RNA by a poly(A) selection with oligo(dT) beads, followed by fragmentation and random priming of mRNA to generate double-stranded cDNA fragments. Subsequently, adaptors were ligated to cDNA fragments, which were then amplified and purified with SPRIselect beads (Beckman Coulter); no size-selection was performed. Size distribution of the final libraries was assessed on a Bioanalyzer with a DNA High Sensitivity kit (Agilent Technologies), and concentration was measured with a Qubit DNA High Sensitivity kit in Qubit 2.0 Fluorometer (Life Technologies). The 18 libraries were pooled by nine and sequenced using NextSeq 500 platform (Illumina) with a read length of 85 nt single-end mode. Libraries for small RNA sequencing were prepared manually with NEB Next multiplex smRNA Library Prep kit (New England Biolabs) from 300 ng of RNA and amplified with 14 PCR cycles. Obtained libraries that passed the QC step, which was assessed on the Agilent Bioanalyzer system (Agilent Technologies), were pooled in equimolar amounts and sequenced on HiSeq 2500 (Illumina) with a read length of 50 nt single-end mode. All the RNA sequencing experiments, including preparation of the libraries, were performed in the GeneCore facility at EMBL, Heidelberg.

### RNA sequencing data analysis

#### Read mapping and filtering

mRNA sequencing quality was assessed by FastQC (version 0.11.5) and MultiQC (version 1.0). Reads were trimmed from adapter sequences and mapped using STAR (version 2.4.2a) to the human reference genome (GRCh38) using default parameters (i.e., the maximal number of mismatches in a read alignment was 10). The gene count matrices were also generated during the alignment using GRCh38.84 annotation; 90.72%–91.87% of reads were mapped for total and polysomal RNA samples; 82.12%–84.94% of reads were mapped for unbound RNA samples. Reads mapping to only one gene were counted.

Small RNA reads were processed by Chimira pipeline (Vitsios and Enright 2015) to obtain information on miRNA changes. To assess small RNA changes, small RNA reads were mapped to a genome reference GRCh38.84 using arguments: “bowtie -n 1 -l 10 -m 100 -k 1 -p 40 ‐‐best ‐‐strata” and counted by “htseq-count ‐‐format sam ‐‐nonunique all” using the gencode.v32.annotation.gtf annotation file.

### Gene expression analysis

Differential expression was assessed using R package DESeq2 (version 1.22.2) (Love et al. 2014). The count matrix was prefiltered for gene IDs that contained at least four reads in at least three samples. DESeq2 results were further devoid of gene IDs with a nonapplicable adjusted *P*-value for downstream analysis. GSEA was performed by using the ranked list of DESeq2 results with a “stat” column used for ranking.

### Editing events analysis

Obtained BAM files were deduplicated using SAMtools (version 1.7) (Li et al. 2009). Editing events were determined using JACUSA (version 1.2.3) (Piechotta et al. 2017). Analysis was done using triplicates of total RNA sequencing data. The filter's setting for analysis was to filter variant calls in the vicinity of read start/end, splice site and homopolymer sequence, minimum of five reads coverage, consider only AG changes, with respect to the libraries being stranded and minimum read quality 20 (call-2 –a B,S,Y –c 5 –C AG –f B –P RF-FIRSTSTRAND, RF-FIRSTSTRAND q -20). We obtained 308,484 positions passing the set filters (414,473 positions did not pass). Based on the recommendation provided in the JACUSA publication (Piechotta et al. 2017), we then set the passing statistics threshold to 2.00 to put more stringent requirements on our edited position calls. Piechotta et al. (2017) recommend 1.56 statistics for duplicates. We set the threshold more stringently following the recommendation in their Supplemental Figure 9; 10,003 nt positions met these statistical criteria. We further limited our analysis to 8664 positions that were edited only in Huh7.5 wt samples and not in Huh7.5 ADAR1 KO samples.

## Software and databases used

FastQC (version 0.11.5) (Andrews 2010)
MultiQC (version 1.0) (Ewels et al. 2016)
STAR (version 2.4.2a) (Dobin et al. 2013)
SAMtools (version 1.7) (Li et al. 2009)
JACUSA (version 1.2.3) (Piechotta et al. 2017)
Chimira release 1.5 (Vitsios and Enright 2015)
Python (version 3.6)
R version 3.5.2 (2018-12-20)
ggplot2_3.1.1
DESeq2_1.22.2 (Love et al. 2014)
Vennerable_3.1.0.9000
gplots_3.0.1.1
biomaRt_2.38.0 (Smedley et al. 2015)
MATLAB 2020a
WebGestalt 2019 (Liao et al. 2019)
miRTarBase (version 7.0) (Huang et al. 2019)
miRDB (version 6.0) (Chen and Wang 2020)
miRBase (version 22.1) (Kozomara et al. 2019)
Repbase (version 23.11) (Bao et al. 2015)
REDIportal (Mansi et al. 2021)
ADeditome (Wu et al. 2021)

## DATA DEPOSITION

The sequencing reads have been deposited in the European Nucleotide Archive (ENA) at EMBL-EBI under accession number PRJEB50933. Information on individual library type and its ID is in Supplemental Table S17.

## SUPPLEMENTAL MATERIAL

Supplemental material is available for this article.

## Figures and Tables

**Kristina Roučová:**
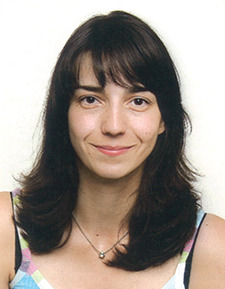


## MEET THE FIRST AUTHOR

***Meet the First Author(s)* is an editorial feature within *RNA*, in which the first author(s) of research-based papers in each issue have the opportunity to introduce themselves and their work to readers of *RNA* and the RNA research community. Kristina Roučová is the first author of this paper, “Loss of ADAR1 protein induces changes in small RNA landscape in hepatocytes.” Kristina is a PhD student at the Laboratory of RNA Biochemistry, Faculty of Science, Charles University in Prague, Czech Republic. The main focus of her research is on RNA editing and analysis of the translatome.**

**What are the major results described in your paper and how do they impact this branch of the field?**

A major result of our work is the establishment of an *ADAR1* KO cell line derived from differentiated human hepatocytes. This broadens the spectrum of *ADAR1* KO cell lines available to us and other researchers to conduct research on, notably if the studied problem has specific demands on the tissue type. We provide a detailed characterization of multiple areas of changes in ADAR1 KO Huh7.5. At the mRNA level, we systematically analyzed changes in transcriptome and translatome. We describe alterations of the small RNA landscape in the *ADAR1* KO cell line, namely, in miRNA, snoRNA, and Y RNA levels. Furthermore, we identified Huh7.5 specific A-to-I editing events at both small RNA and mRNA levels and assessed the A-to-I editing connection to the transcriptome and translatome changes. We found that Huh7.5 hepatocytes are more sensitive to interferon than HEK293 cells, and Huh7.5 ADAR1 KO even more so. Huh7.5 ADAR KO cells also show low-level intrinsic activation of the interferon-stimulated genes. This may suggest why the liver is the most affected tissue in the *ADAR1* KO mice. Last but not least, the Huh7.5 cell line is an important model in virological research. Generation of Huh7.5 ADAR1 KO thus paves the way for new research aimed at understanding how hepatotropic viruses interact with the cell antiviral defense machinery, of which ADAR1 is an important player.

**What led you to study RNA or this aspect of RNA science?**

Throughout my undergraduate studies, I found the impressive versatility of RNA undervalued by my fellow students. In some minds, it was reduced to an overlooked middle man between a gene and a protein product. Instead, I saw an essential cogwheel that connects everything and makes things happen in the cell. RNA can serve as an information carrier, a scaffold for assembling a macromolecular complex, or as an executive molecule of a certain function. It is subjected to countless regulations and modifications. RNA editing, as one of the modifications, can alter all of the mentioned RNA's roles. I think it is all these possibilities that make RNA research a fascinating field to study.

**During the course of these experiments, were there any surprising results or particular difficulties that altered your thinking and subsequent focus?**

The most challenging part of this project was the actual acquirement of the KO cell line. I think everybody can relate to the frustration of a prolonged optimization of your protocol, especially when you are not sure if what you are trying to achieve can realistically be done. We knew from other groups’ work with mouse models that the loss of ADAR1 protein causes many problems that are incompatible with the viability of the animal, and the liver belongs to the most affected tissues. In the mouse models, the lethality was overcome by simultaneously knocking out a downstream signaling molecule of the dsRNA sensing pathway (MAVS). Slightly reluctantly, we were prepared to opt for the double knockout solution, which we inevitably did after a number of attempts and failures to create an ADAR1 KO Huh7.5 cell line. With MAVS KO, we verified the functionality of our CRISPR system, so at that point we were almost convinced the double KO was the only way. Luckily, I kept performing more optimization versions of the *ADAR1* KO-only protocol in parallel with the double KO plan, and it actually paid off. So, it was a kind of double alteration of thinking, where we almost gave up on the plain *ADAR1* KO, but eventually succeeded and realized that it can be done.

**Are there specific individuals or groups who have influenced your philosophy or approach to science?**

I still remember a piece of advice that was given to us by David Staněk during our undergraduate studies: “Don't be afraid to kill your babies.” It is meant to make you critically rethink your hypothesis based on your results. Hopefully, in this way you can drop a pointless topic or a direction of research before it drains you physically and emotionally, not to mention resources and career development. On the other hand, a scientist should be perseverant and not easily deterred by obstacles and difficulties. In that, I am greatly inspired by my supervisor Martin Pospíšek and my advisor Vladimír Beneš. For me, they embody the saying, “There is a solution to every problem.” Their optimism and enthusiasm helped me through various troubleshooting, inconclusive results stretches and learning a considerable amount of new methods during my PhD studies. I feel these two notions are tugging you in the opposite direction, and finding the right balance is the basis to a healthy approach to science.
